# Florida *Shock Anxiety Scale* para Portadores de Cardioversor-Desfibrilador Implantável – Valorizando o Psicossocial

**DOI:** 10.36660/abc.20200262

**Published:** 2020-05-22

**Authors:** Eduardo Arrais Rocha, Ieda Prata Costa

**Affiliations:** Faculdade de Medicina Universidade Federal do Ceará FortalezaCE Brasil Faculdade de Medicina da Universidade Federal do Ceará – Cardiologia, Fortaleza, CE – Brasil; Centro de Arritmia do Ceará FortalezaCE Brasil Centro de Arritmia do Ceará, Fortaleza, CE – Brasil

**Keywords:** Desfibriladores Implantáveis, Qualidade de Vida, Impacto Psicossocial, Ansiedade, Transtornos Psicofisiológicos

Este trabalho apresenta uma importante escala, já em versão brasileira, com validação em nossa população, que permite avaliar o nível de ansiedade relacionada ao cardioversor-desfibrilador implantável (CDI) e aos choques aplicados pelo dispositivo.^1^

Esta escala, como bem ressaltada no trabalho de Silva et al.,^1^ não foi idealizada para avaliar aspectos da adaptação do paciente com o seu CDI ou os impactos desse dispositivo na qualidade de vida do indivíduo, existindo outra escala apropriada para este fim, como a *Florida Patient Acceptance Survey* (FPAS).^2^

Este artigo sinaliza a importância da valorização dos aspectos psicossociais dos portadores de CDI, muitas vezes, relevados a planos relevados a planos secundários. Alguns artigos já demonstraram o impacto negativo que o CDI pode trazer na vida dos pacientes, mesmo sem a ocorrência de terapias.^3-5^

Entretanto, sabe-se do indiscutível benefício que os CDI promovem em diversos contextos clínicos.^6^ Portanto, uma abordagem psicossocial deveria obrigatoriamente fazer parte dos ambulatórios de arritmia e marca-passo, tendo agora disponível em nosso meio uma ferramenta para tal análise.

Manzoni et al.^7^ analisaram 60 estudos e avaliaram o nível de ansiedade, depressão, qualidade de vida relacionada à saúde, síndrome do estresse pós-traumático e transtornos psiquiátricos em portadores de CDI. Eles concluíram que há uma grande heterogeneidade metodológica nos testes psicológicos utilizados.

Isto tem dificultado a análise dos dados e, consequentemente, as estratégias para a diminuição desse impacto. Vários fatores podem influenciar o grau de ansiedade e muitos não foram abordados nesses estudos, como as diferentes características demográficas, as condições clínica e psicológica pré-implante e o número de choques, se apropriados ou não.^7^

Uma subanálise do estudo *Multicenter Automatic Defibrillator Implantation Trial-Reduce Inappropriate Therapy* (MADIT-RIT), que utilizou a escala *Florida Shock Anxiety* Scale (FSAS), concluiu que 2 ou mais choques, apropriados ou inapropriados, e o maior número de episódios de *anti-tachycardia pacing* (ATP) inapropriados estariam associados a maior grau de ansiedade, em um acompanhamento de 9 meses. Por essa análise, sugeriu-se que alterações na programação do CDI podem alterar o grau de ansiedade ao diminuir o número de choques e ATP inapropriados.^8^

Outro tópico relevante refere-se aos cuidados com as programações do CDI. As sociedades da especialidade têm publicações sugerindo a forma ideal de programação, conforme cada fabricante. Essas recomendações devem ser implementadas tentando-se minimizar as terapias de choque, que podem elevar o grau de ansiedade e estar relacionadas a pior prognóstico.^9,10^

A valorização das terapias com ATP (estimulação antitaquicardia), o aumento no tempo ou a programação de um maior número de batimentos para a detecção das arritmias ventriculares sustentadas são extremamente importantes.

A programação do CDI em pacientes com com cardiopatia chagásica (CCH) deve ser diferente da realizada para outras patologias. Na CCH, observa-se um maior número de terapias, em diferentes zonas de detecção, com repercussões clínicas distintas, portanto, merecendo uma abordagem mais individualizada e especializada em centros de referência.

A correta programação das funções de discriminação das arritmias ventriculares, em relação às arritmias supraventriculares, ou mesmo para a identificação de ruídos que possam deflagrar terapias inapropriadas, merece especial atenção. Sabe-se que existem diversos fabricantes com particularidades de programações, mas todas devem ser de conhecimento obrigatório do especialista. A própria escolha do dispositivo com maior duração de bateria, evitando-se trocas precoces, também é um fator importante de proteção psicossocial.

Por fim, parabenizamos os autores pela importante contribuição ao tema no cenário nacional e pelo rigor científico adotado. A implementação da escala FSAS, traduzida e validada por Silva et al., para análise do grau de ansiedade em portadores de CDI auxiliará no tratamento psicossocial destes pacientes.
