# Supplementary material for: Florida Shock Anxiety Scale para Portadores de Cardioversor-Desfibrilador Implantável – Valorizando o Psicossocial
Source: Arq Bras Cardiol. 2020 May 22;114(5):773–4. [Article in Portuguese] doi: 10.36660/abc.20200262 (PMC8387010; doi:10.36660/abc.20200262)
Supplement: Translation

# Translation: Florida Shock Anxiety Scale for Patients with Implantable Cardioverter-Defibrillator - Appreciating the Psychosocial Aspects

**Authors:** Eduardo Arrais Rocha, Ieda Prata Costa

**Affiliations:** Brazil Faculdade de Medicina da Universidade Federal do Ceará – Cardiologia, Fortaleza, CE – Brazil; Brazil Centro de Arritmia do Ceará, Fortaleza, CE – Brazil

**Keywords:** Defibrillators, Implantable, Quality of Life, Psychosocial Impact, Anxiety, Psychophysiologic Disorders

The published study provides an important scale, already in its Brazilian Portuguese version and that has been validated for our population, which allows assessing the level of anxiety related to the presence of the Implantable Cardioverter-Defibrillator (ICD) and the shocks applied by the device.^1^

This scale, as well emphasized in the study by Silva et al.,^1^ was not designed to assess aspects of the patient’s adaptation to the ICD, or even its impacts on patient quality of life, and there is another scale that is appropriate for this purpose, such as the FPAS - Florida Patient Acceptance Survey.^2^

The article reminds us of the importance of appreciating the psychosocial aspects of patients with an ICD that are often relegated to a secondary plan. Several articles have demonstrated the negative impact that the presence of the ICD, even without therapies, can have on these patients’ lives.^3-5^

However, we know about the undeniable clinical benefits they bring in different clinical contexts.^6^ Therefore, a psychosocial approach should be part of the arrhythmia and pacemaker outpatient clinics, considering we now have a tool for such analysis available in our country.

Manzoni et al.^7^ analyzed sixty studies and assessed the level of anxiety, depression, health-related quality of life, post-traumatic stress syndrome and psychiatric disorders in patients with ICD. They concluded there is a large methodological heterogeneity in the psychological tests used.

This has made data analysis difficult and, consequently, has hindered strategies to reduce this impact. Several factors can influence the degree of anxiety and are not fully addressed in these studies, such as the different demographic characteristics, the pre-implantation clinical and psychological status and the number of shocks, whether appropriate or not.^7^

A subanalysis of the MADIT-RIT study, which used the FSAS (Florida Shock Anxiety Scale) scale, concluded that >2 appropriate or inappropriate shocks and a higher number of inappropriate Anti-Tachycardia Pacing (ATP) episodes were associated with a greater degree of anxiety, during a 9-month follow-up. Therefore, it has been suggested that changes in the ICD programming may alter the degree of anxiety by reducing the number of inappropriate shocks and ATPs.^8^

Another relevant topic concerns the care with the ICD programming. The Specialty Societies have publications suggesting the ideal form of programming, according to each manufacturer. The implementation of these recommendations must be carried out in an attempt to minimize shock therapies, which can increase the degree of anxiety and be associated with a worse prognosis.^9,10^

The valorization of therapies with ATP (anti-tachycardia stimulation), the increase in time or the programming of a higher number of beats for the detection of sustained ventricular arrhythmias are extremely important.

The programming of patients with Chagas cardiopathy is not possible, as seen in other pathologies, as they have a greater number of therapies, in different detection zones, with different clinical implications, therefore deserving a more individualized and specialized approach in referral centers.

The correct programming of ventricular arrhythmia discrimination functions, in relation to supraventricular arrhythmias, or even for the identification of noises that may trigger inappropriate therapies, deserve special attention. We know there are several manufacturers with certain peculiarities in their programming, which are mandatory for the specialist’s knowledge. The choice of the device that has the longest battery life, preventing early battery change, is also an important factor in the context of psychosocial protection.

Finally, we congratulate the authors for their important contribution to the topic in the national scenario and for the scientific thoroughness adopted in the study. The implementation of this scale, translated and validated by Silva et al. to analyze the degree of specific anxiety in patients with ICDs will help in the psychosocial treatment of these patients.
